# Pterostilbene restores mitochondrial retrograde signaling to activate protective stress responses in Parkinson’s disease

**DOI:** 10.3389/fnagi.2025.1692777

**Published:** 2025-12-12

**Authors:** Fanshuai Zeng, Wei Li, Liyue Yang, Lu Yao, Xinna Wang

**Affiliations:** 1 Department of Neurology, Affiliated Hospital of Changchun University of Chinese Medicine, Changchun, Jilin, China; 2 Department of Pediatric, Affiliated Hospital of Changchun University of Chinese Medicine, Changchun, Jilin, China; 3 Department of Encephalopathy, Changchun Hospital of Traditional Chinese Medicine, Changchun, Jilin, China; 4 Department of Physiology and Pathophysiology, School of Basic Medical Sciences, Xi'an Jiaotong University Health Science Center, Xi'an, Shaanxi, China

**Keywords:** pterostilbene, Parkinson’s disease, mitochondrial retrograde signaling, dopaminergic neurons, neuroprotection, polyphenols, cellular resilience

## Abstract

Parkinson’s disease (PD) is the selective demise of dopaminergic neurons in the substantia nigra. Conventional neuroprotective strategies based on exogenous antioxidants have shown minimal clinical efficacy. Emerging evidence suggests that neuronal loss in PD may stem not only from direct mitochondrial damage but, more critically, from the failure of an intrinsic “early-warning system”—the mitochondrial retrograde signaling (MRS) pathway—impairing the nucleus’s ability to launch timely protective responses. This review repositions pterostilbene, a bioavailable dietary polyphenol, from a simple antioxidant to a “signal fidelity enhancer” that supports mitochondria-to-nucleus communication. By stabilizing mitochondrial function and modulating stress-sensing pathways, pterostilbene may restore MRS integrity and promote activation of endogenous defense mechanisms such as the mitochondrial unfolded protein response (UPRmt). The article advocates a paradigm shift in nutritional neuroprotection: from passive supplementation toward reinforcing the neuron’s intrinsic capacity for self-maintenance and resilience.

## 1 Introduction: the unaddressed dimension of “resilience” in Parkinson’s disease therapy

Parkinson’s disease (PD), the second most prevalent neurodegenerative disorder after Alzheimer’s disease, is characterized by motor symptoms—resting tremor, rigidity, bradykinesia, and postural instability—and debilitating non-motor symptoms including hyposmia, sleep disturbances, depression, and cognitive decline (Tanner and Ostrem, 2024). Current treatments such as levodopa and dopamine agonists provide effective symptomatic relief in early stages but fail to halt or slow the progressive loss of dopaminergic neurons in the substantia nigra pars compacta (SNc) (Backman et al., 2025). As PD advances, patients face motor fluctuations and dyskinesias, leading to a severe decline in quality of life and imposing significant burdens on families and healthcare systems (Fu et al., 2024). Thus, developing true neuroprotective strategies remains an urgent challenge in modern neurology (Meissner et al., 2011).

Mitochondrial dysfunction has been identified as the initiating event in both sporadic and familial PD (Borsche et al., 2021). The discovery that MPTP, a contaminant in synthetic heroin (Narmashiri et al., 2022), selectively destroys SNc neurons by inhibiting mitochondrial respiratory chain complex I via its toxic metabolite MPP^+^, highlighted this link (Delp et al., 2021). Genetic studies further corroborated this finding by identifying mutations in PINK1 and Parkin genes, which regulate mitochondrial quality control processes like mitophagy (Li et al., 2023). This convergence of evidence from toxicology and genetics points to PD being fundamentally a “mitochondriopathy,” where protecting mitochondria equates to protecting dopaminergic neurons.

Initial neuroprotective strategies focused on exogenous antioxidants like vitamin E and coenzyme Q10 due to the role of mitochondrial dysfunction in generating reactive oxygen species (ROS) (He et al., 2024). However, these approaches failed in large-scale clinical trials, showing no significant benefits in slowing disease progression (Robinson et al., 2006; Ahmadi et al., 2024). These failures prompted a reassessment of therapeutic paradigms. Rather than passively suppressing oxidative stress, enhancing the cell’s own endogenous defense and repair systems offers a more promising approach. This involves bolstering cellular resilience—the intrinsic capacity of cells to withstand stress and maintain homeostasis.

Emerging evidence suggests that pterostilbene, a natural dimethylated analog of resveratrol found in blueberries, not only acts as a dietary antioxidant but also modulates mitochondrial homeostasis and stress signaling. Its superior bioavailability, metabolic stability, and blood brain barrier permeability enable effective CNS concentrations following oral administration (Wang and Sang, 2018). Furthermore, pterostilbene potentiates the mitochondrial unfolded protein response (UPRmt), which clears misfolded proteins and maintains organelle function (Suarez-Rivero et al., 2022). By amplifying this endogenous surveillance system, pterostilbene acts as a “signal fidelity enhancer,” reinforcing neurons’ capacity to detect, respond to, and recover from mitochondrial stress before irreversible damage occurs. This mechanism aligns with hormetic neuroprotection, where mild stressors activate adaptive resilience pathways. Given its dual action on mitochondrial energetics and nuclear stress-responsive transcription, supported by robust pharmacokinetics, pterostilbene represents a compelling candidate for early intervention in PD, particularly in prodromal stages where mitochondrial dysfunction precedes overt neuronal loss.

## 2 Mitochondrial dysfunction in Parkinson’s disease: the engine of neurodegeneration

### 2.1 The unique vulnerability of dopaminergic neurons

The selective vulnerability of SNc dopaminergic neurons to mitochondrial insults is not coincidental; it is a direct consequence of their unique and demanding physiology (Zampese et al., 2022; Guzman et al., 2010).

1. Exceptionally High Bioenergetic Demand: A single SNc dopaminergic neuron possesses one of the most extensive axonal arbors in the human brain—spanning up to several meters in total length and forming hundreds of thousands of synapses. Sustaining ionic gradients and neurotransmitter release across this vast network imposes an immense, continuous demand for ATP, which is met almost exclusively through mitochondrial oxidative phosphorylation (Hou et al., 2025).
2. Intrinsic Oxidative Burden from Dopamine Metabolism: Dopamine itself is a source of endogenous oxidative stress. When not safely sequestered into synaptic vesicles, cytosolic dopamine can undergo auto-oxidation or enzymatic degradation by monoamine oxidase, generating reactive oxygen species (ROS) and neurotoxic quinones. Consequently, these neurons operate in a chronically pro-oxidant environment.(Segura-Aguilar et al., 2014).
3. Calcium-Dependent Pacemaking and Mitochondrial Stress: Unlike many other neurons, SNc dopaminergic neurons rely on L-type voltage-gated calcium channels to sustain their autonomous pacemaking activity. This results in sustained elevations in cytosolic calcium, which mitochondria must buffer. Chronic calcium loading predisposes mitochondria to permeability transition pore opening, bioenergetic failure, and further ROS production (Zampese and Surmeier, 2020).

Together, these three interrelated features create a state of heightened dependence on—and susceptibility to—mitochondrial integrity, rendering SNc dopaminergic neurons exquisitely vulnerable to mitochondrial dysfunction.

### 2.2 The cascade of damage: from complex I to cell death

**Figure 1:**
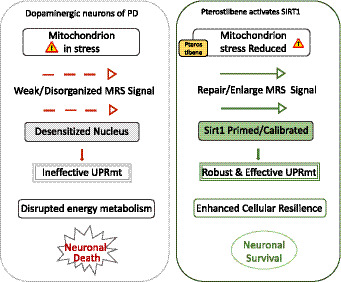
A schematic overview of the core role of mitochondrial dysfunction in the pathogenesis of Parkinson’s disease. Initial insults targeting Complex I trigger a devastating cascade of energy failure, oxidative stress, membrane potential collapse. This primary damage is amplified by failed quality control systems (mitophagy), leading to the accumulation of toxic protein aggregates and chronic neuroinflammation, which collectively drive the progressive loss of dopaminergic neurons.

In PD pathophysiology, mitochondrial damage propagates through a classic feed-forward loop. The initial insult, whether from environmental toxins (such as MPP^+^ or rotenone) or genetic defects, frequently targets mitochondrial respiratory chain complex I. Inhibition of complex I immediately triggers a triad of catastrophic events: (1) a sharp decline in ATP production, leading to an energy crisis; (2) dysfunction in the electron transport chain, causing electrons to leak and generate massive amounts of superoxide radicals (ROS); and (3) a drop in the mitochondrial membrane potential (ΔΨm), disrupting mitochondrial integrity (Subramaniam and Chesselet, 2013). This initial damage initiates a vicious cycle, including oxidative damage to mitochondrial DNA (mtDNA), imbalanced mitochondrial dynamics (excessive fission, impaired fusion), and ultimately, the activation of apoptotic cell death pathways (Alqahtani et al., 2023; Figure 1).

### 2.3 Failure of quality control: the breakdown of mitophagy

Healthy cells employ a sophisticated quality control process called mitophagy to selectively eliminate damaged mitochondria. This process is orchestrated by the PD-associated proteins PINK1 and Parkin. Upon mitochondrial damage and loss of membrane potential, the kinase PINK1 stabilizes on the outer mitochondrial membrane, where it recruits and activates the E3 ubiquitin ligase Parkin. Parkin then ubiquitinates various outer membrane proteins, creating a “tag” that is recognized by autophagy receptors, leading to the engulfment of the damaged mitochondrion by an autophagosome and its subsequent degradation in the lysosome (Pickrell and Youle, 2015). In PD, whether due to genetic mutations or oxidative stress-induced inactivation of PINK1/Parkin, this crucial quality control system fails. This failure allows dysfunctional, ROS-spewing mitochondria to accumulate, continuously poisoning the neuron from within and driving disease progression.

## 3 Mitochondrial retrograde signaling (MRS): the cell’s forgotten “early-warning system”

While mitophagy acts as the “cleanup crew” for damage that has already occurred, a more proactive and upstream defense mechanism exists: the mitochondrial retrograde signaling (MRS) pathway. It can effectively reprogram cellular metabolism before irreversible damage accumulates. Emerging evidence suggests MRS may orchestrate cross-talk with other quality control pathways, creating a tiered defense network.

### 3.1 Defining MRS: distress communication from mitochondria to nucleus

MRS encompasses a set of signaling pathways that originate in the mitochondria and travel to the nucleus to alter the expression of nuclear genes. It is the primary mechanism by which mitochondria communicate their functional status—be it metabolic stress, proteotoxic stress, or damage—to the cell’s command center, thereby initiating adaptive and protective programs (Cardamone et al., 2018). The targeted defect of MRS would damage the balance mechanism of oxidative phosphorylation, ultimately leading to obstacles in the cell metabolic process, and *in vivo*, it can be blocked by drugs to integrate the stress response (Walker et al., 2025). In contrast to the “elimination” function of mitophagy, the core purpose of MRS is “adaptation and repair.”

### 3.2 The key pathway: the mitochondrial unfolded protein response (UPRmt)

In mammalian cells, the best-characterized MRS pathway is the Mitochondrial Unfolded Protein Response (UPRmt) (Kirstein-Miles and Morimoto, 2010). This pathway is activated by an accumulation of misfolded or aggregated proteins within the mitochondrial matrix. The signaling cascade involves the transcription factor ATF5 (ATFS-1 in *C. elegans*). In the process of UPRmt, various components are activitated to enhance the mitochondria-nucleus retrograde signaling, including ATF-4, ATF-5, etc. However, under stress, its mitochondrial import is blocked, leading to its accumulation in the cytosol and subsequent translocation to the nucleus (Keerthiga et al., 2021). Within the nucleus, ATF5 collaborates with other transcription factors like CHOP to activate the transcription of a battery of protective genes, including:

1. Mitochondrial chaperones (e.g., HSP60): to assist in protein refolding.
2. Mitochondrial proteases: to degrade irreparable proteins.
3. Antioxidant enzymes: to neutralize ROS.
4. Enzymes for metabolic remodeling: to adapt cellular metabolism to the stress.

This coordinated response aims to restore mitochondrial proteostasis before the damage becomes irreversible (Das et al., 2020; Oliveira and Hood, 2018).

### 3.3 The “warning system failure” hypothesis in PD

In the early stages of PD, the MRS and UPRmt pathways become dysfunctional. Chronic, low-grade oxidative stress and protein damage may either exhaust the response capacity of the UPRmt or render the signaling pathway itself “desensitized.” This allowed the disease to gain the upper hand once again. This failure of the early-warning system is catastrophic. It means the neuron loses its window of opportunity for early self-repair and adaptation. It is forced to passively accumulate damage until the point of no return, where the only remaining options are the drastic measures of mitophagy or apoptosis (Lin et al., 2018). Therefore, identifying molecules that can recalibrate and amplify this warning signal represents a highly attractive and novel neuroprotective strategy.

## 4 Pterostilbene: a pharmacologically optimized polyphenol for neuroprotection

Why, out of the myriad of plant polyphenols, is pterostilbene the ideal candidate for restoring MRS? The answer lies in its unique chemical structure, which confers superior pharmacological properties.

### 4.1 The “resveratrol problem” and the “Pterostilbene solution”

Resveratrol is arguably the most studied neuroprotective polyphenol, yet its clinical translation has been largely unsuccessful. The primary obstacle is its abysmal pharmacokinetic profile. Pterostilbene, as a naturally occurring dimethoxylated derivative of resveratrol, elegantly overcomes these limitations. From the perspective of chemical structure, Pterostilbene’s two methoxy groups (-OCH₃) replace two of resveratrol’s hydroxyl groups (-OH). From the perspective of pharmacological advantages, Pterostilbene has demonstrated excellent bioavailability and metabolic stability. The methoxy groups increase the molecule’s lipophilicity, facilitating its absorption across the intestinal wall. Studies indicate that the bioavailability of pterostilbene is approximately 80%, compared to only ~20% for resveratrol. Pterostilbene exhibits superior oral bioavailability and higher systemic exposure of both parent compound and metabolites compared to resveratrol, suggesting potentially enhanced *in vivo* biological activity at equimolar doses (Kapetanovic et al., 2011). This decisive pharmacokinetic advantage means that pterostilbene can achieve higher and more sustained therapeutic concentrations in the body at lower doses, a critical prerequisite for any viable clinical candidate. Simultaneously, the hydroxyl groups are the primary targets for rapid phase II metabolism (glucuronidation and sulfation). The protective methoxy groups make pterostilbene much less susceptible to metabolic clearance. After pharmacokinetic testing in SD rats, Pterostilbene was found to be eliminated slowly *in vivo*, and its metabolic stability was significantly better than that of resveratrol, mainly due to its dimethoxy structure, which reduced the sensitivity to glucuronidation and sulfation metabolism. Although resveratrol may reach a higher peak plasma concentration (Cmax) more quickly after oral administration, its metabolism is extremely unstable, it is cleared rapidly, and has low bioavailability and it is difficult to improve through formulation or dose optimization (Wang and Sang, 2018). Therefore, Pterostilbene outperforms resveratrol in terms of oral exposure, duration, and formulation response, making it a more promising candidate molecule for development, see Table 1 for details.

**Table 1:** Pharmacokinetic comparison of resveratrol and pterostilbene.

Parameter	Resveratrol	Pterostilbene	Advantage summary
Oral bioavailability (%)	~20%	~80%	~4-fold increase
Plasma Half - life (t½)	~14 min	~105 min	~7.5-fold prolongation
Lipophilicity (LogP)	3.1	4.1	Higher, facilitating transmembrane transport
BBB permeability	Low	Moderate	Superior central delivery

### 4.2 Established neuroprotective mechanisms: a solid foundation

Before proposing a novel mechanism, it is crucial to establish that pterostilbene possesses a solid foundation of relevant biological activities. Pterostilbene is a potent activator of the Nrf2 pathway, the master regulator of endogenous antioxidant defenses. It also robustly inhibits the NF-κB signaling pathway, thereby suppressing microglial activation and the production of pro-inflammatory cytokines(Zhu et al., 2020), which confirms its pharmacological effects of antioxidant and anti - inflammatory. Pterostilbene is a powerful activator of SIRT1 (Sirtuin 1), a critical energy sensor and longevity-associated protein. SIRT1, through its deacetylase activity, regulates a wide array of cellular processes, including mitochondrial function, autophagy, and stress responses (Monceaux et al., 2022). From the perspective of the function of Pterostilbene in activating SIRT1, it can be seen that it has great medicinal potential. Direct evidence shows that Pterostilbene can inhibit ROS on mitochondria, promote mitochondrial biogenesis, and inhibit mitochondria-mediated apoptosis (He et al., 2018). This strongly suggests that Pterostilbene seems to be a natural weapon against “mitochondrial diseases.”

### 4.3 Regulation mechanism of blood–brain barrier permeability

For any central nervous system (CNS) therapeutic, the ability to efficiently cross the blood–brain barrier (BBB) is non-negotiable. Pterostilbene’s enhanced lipophilicity gives it a distinct advantage. In animal model experiments of cerebral hemorrhage, it was found that Pterostilbene could alleviate the damage to the blood–brain barrier function and cerebral edema, verifying its ability to cross the BBB and exert central effects (Wu et al., 2023). In both *in vivo* and *in vitro* experiments of cerebral ischemia/reperfusion, it has been demonstrated that Pterostilbene can increase cerebral microcirculation and improve blood–brain barrier leakage (Yang et al., 2023).

## 5 The central hypothesis: Pterostilbene as a “signal Fidelity enhancer” for MRS

Pterostilbene functions not as a passive antioxidant barrier, but as an active modulator of mitochondrial–nuclear communication that enhances the fidelity of stress signaling and reinforces endogenous cytoprotective pathways.

### 5.1 Integrating the evidence: synergy between SIRT1 and UPRmt

**Figure 2:**
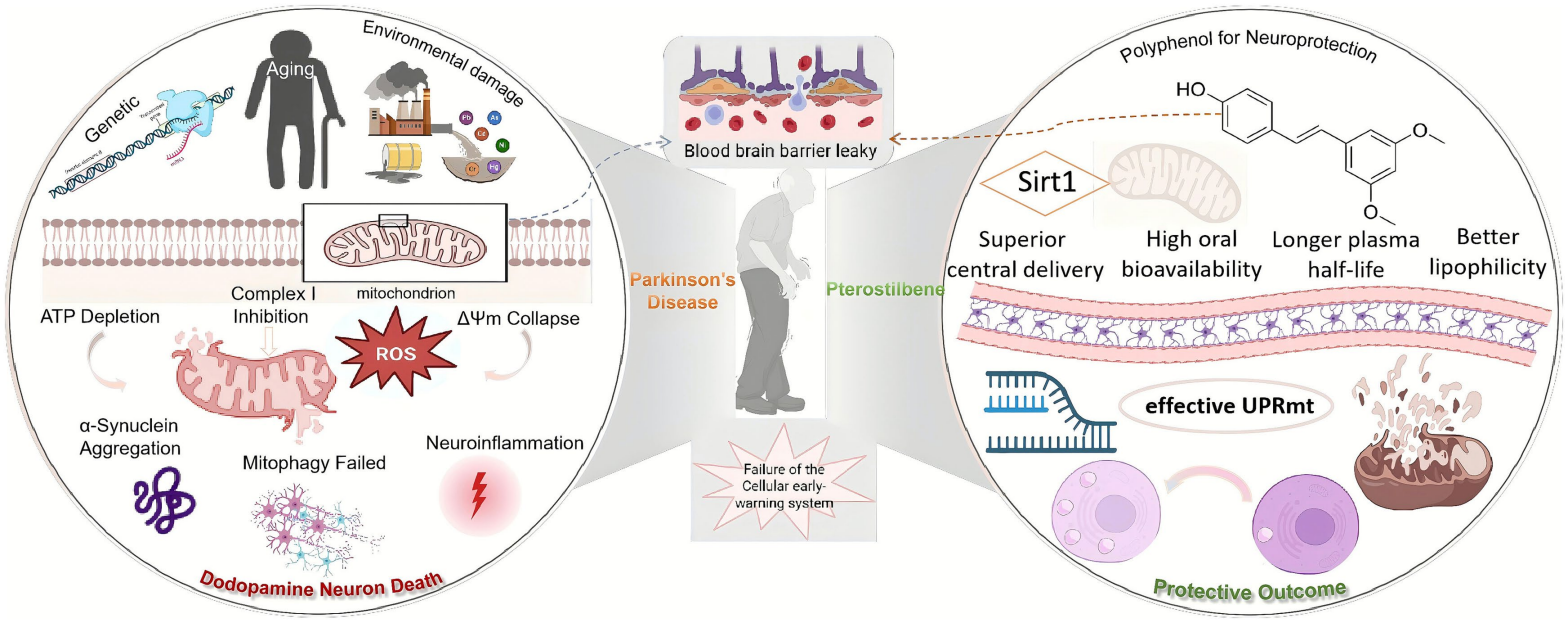
Pterostilbene modulates mitochondrial retrograde signaling to elicit neuroprotective stress responses in Parkinson’s disease. In pathological states, mitochondrial–nuclear communication is disrupted, leading to cell death. Pterostilbene enhances neuronal resilience by acting as a homeostatic regulator that restores cellular sensitivity to mitochondrial distress signals.

In PD neurons, mitochondria are under chronic stress, emitting weak or disorganized retrograde signals. The nuclear response machinery is desensitized, and the UPRmt is not effectively activated. After the medication is ingested into the body, Pterostilbene crosses the BBB and enters the neuron, acting as a potent SIRT1 activator. Subsequently, the activation of SIRT1 leads to the deacetylation and modulation of numerous stress-response transcription factors (such as FOXO, PGC-1α). This action effectively primes or calibrates the nuclear defense system, making it more receptive and sensitive to incoming stress signals from the mitochondria. Crucially, SIRT1 activity has been shown to be essential for a robust and effective UPRmt (Suarez-Rivero et al., 2022; Mouchiroud et al., 2013). When the stressed mitochondria now send a retrograde signal (e.g., via ATF5 translocation), the “primed” nucleus can respond earlier, more strongly, and more accurately, launching a full-scale, efficient UPRmt program. A battery of endogenous chaperones, proteases, and antioxidant enzymes are transcribed and translated. Damaged mitochondria are repaired, proteostasis is restored, and the cell successfully resolves the stress before it becomes irreversible. Furthermore, the Protective Outcome was formed (Figure 2).

### 5.2 From passive defense to actively enhanced resilience

This model fundamentally redefines our understanding of how polyphenols can exert their effects. It moves beyond a simple 1:1 stoichiometric battle of antioxidants against ROS, such as curcumin (Huang et al., 2013) and EGCG (Karthikeyan et al., 2017). Instead, pterostilbene acts catalytically, leveraging the cell’s own sophisticated defense network. Rather than fighting stress on behalf of the cell, pterostilbene reinstates the cell’s intrinsic stress-sensing mechanisms and reengages its transcriptional command network, enabling autonomous defense and repair. This is the very essence of enhancing cellular resilience.

## 6 From mechanism to medicine: translating Pterostilbene for Parkinson’s disease

While pterostilbene has shown neuroprotective effects in various PD rodent models, its path from laboratory to clinical application is not yet complete. Critically, no dedicated clinical trials have yet evaluated pterostilbene in PD patients. Nevertheless, clinical trials in aging and non-alcoholic fatty liver disease suggest that no serious adverse events reported (Dellinger et al., 2023; Majeed et al., 2020). Its ability to restore mitochondrial–nuclear signaling and boost endogenous stress resilience aligns with several unmet needs:

### 6.1 Addressing the lack of disease-modifying therapies

Current pharmacotherapies for Parkinson’s disease (PD), such as levodopa, provide symptomatic relief but fail to modify the underlying neurodegenerative trajectory. In contrast, pterostilbene represents a neuroprotective strategy by targeting upstream pathogenic mechanisms—including mitochondrial dysfunction, impaired mitochondrial unfolded protein response (UPRᵐᵗ), and disrupted proteostasis—thereby addressing core drivers of neuronal vulnerability rather than merely alleviating clinical manifestations. To rigorously evaluate its disease-modifying potential, future phase II clinical trials should adopt a biomarker-driven design, incorporating modalities such as magnetic resonance spectroscopy (e.g., NAA/Cr ratio), circulating mtDNA copy number, or plasma markers of mitochondrial stress to objectively assess target engagement and biological impact on disease progression.

### 6.2 Overcoming blood–brain barrier (BBB) penetration challenges

A major obstacle in the clinical development of neuroprotective agents for PD is inadequate CNS bioavailability, with many promising compounds failing to achieve sufficient brain exposure due to poor BBB penetration. Pterostilbene distinguishes itself among nutraceuticals through its high lipophilicity, favorable oral bioavailability, and consistent demonstration of brain penetration in preclinical models, collectively supporting its potential for meaningful CNS engagement. To translate this pharmacokinetic advantage into clinical practice, human studies employing positron emission tomography (PET) imaging or cerebrospinal fluid (CSF) sampling are warranted to quantify brain concentrations at putative neuroactive doses, thereby enabling evidence-based dose selection for subsequent efficacy trials.

### 6.3 Targeting early-stage or prodromal PD

Neuroprotective interventions are most likely to succeed when administered during the early or prodromal stages of PD, prior to extensive and irreversible dopaminergic neuron loss; however, the clinical identification of at-risk individuals remains challenging. Pterostilbene is well-suited for this preventive window due to its favorable safety profile and potential for chronic administration in populations with elevated risk, such as those with REM sleep behavior disorder or pathogenic LRRK2 variants. To evaluate its efficacy in delaying or preventing disease onset, pterostilbene should be integrated into prospective prevention trials enrolling prodromal cohorts, with outcomes anchored in multimodal biomarkers—including digital phenotyping via wearable sensors, objective olfactory testing, and dopamine transporter imaging (DaT-SPECT)—to sensitively capture preclinical disease progression and therapeutic impact.

### 6.4 Integrating Pterostilbene into holistic PD management: combination therapy, systemic benefits, and global accessibility

Pterostilbene’s role as a homeostatic modulator supports its evaluation as an add-on to standard dopaminergic therapy, potentially enhancing neuronal resilience and mitigating levodopa-induced oxidative stress without pharmacological interference. Its systemic actions—improving metabolic health, reducing inflammation, and protecting peripheral neurons—may also address non-motor symptoms linked to mitochondrial dysfunction in the gut and autonomic nervous system. We acknowledge that this review is primarily based on animal experiments and *in vitro* studies, and that the evidence base has certain limitations. Accordingly, clinical trials should include non-motor endpoints such as MDS-UPDRS Part I and autonomic assessments alongside motor outcomes. Given its natural origin, oral bioavailability, and low-cost production, pterostilbene represents a scalable candidate for global deployment; pragmatic effectiveness trials in diverse healthcare settings, developed in partnership with public health agencies, would help bridge the gap between neuroprotective promise and real-world accessibility.

## 7 Conclusion

Neuroprotection research in Parkinson’s disease is at a crossroads, with traditional antioxidant strategies having reached their limits. This review has systematically proposed a novel therapeutic paradigm: a shift from passive defense to the active enhancement of endogenous cellular resilience. We have argued that the PD pathology, centered on mitochondrial dysfunction, may be critically driven by a failure of the cell’s “early-warning system”-the mitochondrial retrograde signaling pathway. With its favorable pharmacokinetics and potent bioactivity, pterostilbene emerges as an ideal therapeutic candidate that functions as a “signal fidelity enhancer” to reinstate a robust UPRᵐᵗ via SIRT1 activation, thereby enhancing neuronal stress resilience—a strategy with strong translational potential. Proposing a paradigm shift in nutritional strategies for Parkinson’s disease—moving from exogenous protection toward restoring endogenous regulatory networks to bolster neuronal resilience and self-sustaining capacity.
